# The perception of Mandarin speech conveying communicative functions in Chinese heroin addicts

**DOI:** 10.1371/journal.pone.0299331

**Published:** 2024-02-23

**Authors:** Puyang Geng, Ningxue Fan, Rong Ling, Hong Guo, Qimeng Lu, Xingwen Chen

**Affiliations:** 1 Academy of Forensic Science, Shanghai, China; 2 Shanghai Forensic Service Platform, Key Laboratory of Forensic Science, Ministry of Justice, Shanghai, China; 3 Information Security and Social Management Innovation Lab, Shanghai Open University, Shanghai, China; 4 Network Security Team, Public Security Department of Guangxi Province, Nanning, Guangxi, China; Peking University, Institute of Mental Health, CHINA

## Abstract

Drug addiction can cause severe damage to the human brain, leading to significant problems in cognitive processing, such as irritability, speech distortions, and exaggeration of negative stimuli. Speech plays a fundamental role in social interaction, including both the production and perception. The ability to perceive communicative functions conveyed through speech is crucial for successful interpersonal communication and the maintaining good social relationships. However, due to the limited number of previous studies, it remains unclear whether the cognitive disorder caused by drug addiction affects the perception of communicative function conveyed in Mandarin speech. To address this question, we conducted a perception experiment involving sixty male participants, including 25 heroin addicts and 35 healthy controls. The experiment aimed to examine the perception of three communicative functions (i.e., statement, interrogative, and imperative) under three background noise conditions (i.e., no noise, SNR [Signal to Noise Ratio] = 10, and SNR = 0). Eight target sentences were first recorded by two native Mandarin speakers for each of the three communicative functions. Each half was then combined with Gaussian White Noise under two background noise conditions (i.e., SNR = 10 and SNR = 0). Finally, 48 speech stimuli were included in the experiment with four options provided for perceptual judgment. The results showed that, under the three noise conditions, the average perceptual accuracies of the three communicative functions were 80.66% and 38% for the control group and the heroin addicts, respectively. Significant differences were found in the perception of the three communicative functions between the control group and the heroin addicts under the three noise conditions, except for the recognition of imperative under strong noise condition (i.e., SNR = 0). Moreover, heroin addicts showed good accuracy (around 50%) in recognizing imperative and poor accuracy (i.e., lower than the chance level) in recognizing interrogative. This paper not only fills the research gap in the perception of communicative functions in Mandarin speech among drug addicts but also enhances the understanding of the effects of drugs on speech perception and provides a foundation for the speech rehabilitation of drug addicts.

## 1. Introduction

Drugs refer to non-medical narcotics and psychotropic substances that can cause dependency or addiction. Currently, common drugs include heroin, marijuana, cocaine, methamphetamine, ecstasy (i.e., 3,4-methylenedioxymethamphetamine), ketamine, and synthetic cannabinoids. Due to their ability to cause significant damage to human physical and mental health, drug use is strictly prohibited in China and worldwide. According to the 2021 drug report proposed by United Nations Office on Drugs and Crime (UNODC), nearly 300 million people worldwide are addicted to drugs. Even more alarming, a larger number of people are suffering from diseases such as HIV/AIDS, depression, and mental disorders caused by drug use. It is evident that the frightening aspect of drugs is not only their intense addiction that is difficult to overcome but also their severe impact on the physical and mental health of addicts.

### 1.1 Effect of drugs on cognitive processing

Numerous neuroscientific studies conducted on drug addicts have confirmed that drugs can cause severe damage to the brain structure of addicts. Previous studies have shown that gray matter reduction occurs in the frontal lobe, dorsolateral prefrontal cortex, temporal lobe, and cingulate gyrus cortex of opioid addicts (e.g., heroin) [1]. Using imaging measurement techniques such as CT and magnetic resonance imaging, some researchers have found that the brain volume of opioid addicts decreases, the frontal lobe volume is reduced, and the white matter intensity in the frontal lobe increases [2, 3]. Gaudreault et al. [4] found that the brain white matter volume of heroin and cocaine addicts is significantly reduced, with cocaine addicts being more severely affected than heroin addicts. Some studies have also found that brain damage in areas such as the frontal lobe cortex and gray matter increases gradually with prolonged heroin use [5, 6]. In addition, Wang et al. [3, 7] found that the gray matter and white matter density of the frontal lobe cortex and cingulate gyrus were reduced three days after drug detoxification. After one month of detoxification, there was no significant difference in the frontal gyrus between heroin addicts and the control group, but changes in other brain regions such as the right frontal middle gyrus, left cingulate gyrus, and left occipital gyrus still existed. This indicates that there is no sign of recovery in the brain structure of addicts after short-term detoxification.

As a result of the impact of these drugs on the human brain, addicts experience serious cognitive processing disorders, such as schizophrenia [8], attentional bias [9], and decision-making disorders [10]. In addition, studies have found that addicts also have problems with time and emotion processing, which are closely related to speech perception (e.g., speech rate perception and emotion recognition). For example, previous studies have found that the perception of time by heroin and cocaine addicts is shorter than the actual duration [11, 12]; Moreira et al. [13] reviewed medical records and research and found that drug addicts usually overestimate time intervals. Moreira et al. believe that the reason for the time perception disorders in addicts may be that they have higher impulsivity and are more inclined to react quickly. As for emotions, previous studies have found that addicts usually exhibit symptoms such as irritability and negative reinforcement. Some researchers have found that drug addicts have lower activity in processing emotional stimuli [14] or are more likely to be attracted to negative stimuli [3, 15]. May et al. [16] believe that addicts have difficulty understanding their own and others’ behavior and have difficulty regulating their emotions, making them more prone to negative emotions or being attracted to negative stimuli.

### 1.2 Effect of drugs on speech perception

Interpersonal communication is a necessary factor in maintaining physical and mental health and establishing good social relationships. Research has shown that self-esteem and social support can significantly reduce the relapse rate of addicts [17]. In daily life, speech communication is the main method of interpersonal communication, which includes both speech production and perception. Among them, the perception of communicative functions expressed by the speaker (such as interrogative and imperative) not only relates to the success of social communication activities, but may also directly affect interpersonal and social relationships.

Based on the literature review in section 1.1, it can be seen that drug addicts may experience serious cognitive processing disorders, including time and emotion processing disorders closely related to speech perception. So, do drug addicts have difficulties in speech perception? In particular, is there any abnormality in the understanding of communicative functions conveyed through speech in addicts in daily social interactions? For these questions, there is still a lack of systematic research on speech perception in drug addicts, and only a few relevant studies can provide some insights.

Studies have found that drug addicts exhibit psychosis-like symptoms, such as speech illusions. Honey et al. [18] found that ketamine addicts exhibited speech illusions when asked questions about speech stimuli (such as whether the volume had decreased or increased). Mokrysz et al. [19] found that cannabis can cause an increase in the Psychotomimetic States Inventory scores (the higher the score, the more likely it is to exhibit psychosis-like symptoms), and the results of subsequent speech perception experiments showed that cannabis users believed they had heard positive or negative speech under conditions where only white noise was provided. Similarly, Lozano-López et al. [8] found through a review that heroin, methadone, and morphine addicts all reported speech illusions. However, some scholars hold a different view. Fachner believes that cannabis can improve the music perception ability of users and proposed that cannabis can make users more focused on the perception of sound, and enhance the acoustic signal perception ability of addicts to a certain extent [20, 21].

Currently, the reason for the occurrence of speech illusions in drug addicts is still unknown. However, researchers have extensively discussed the causes of speech illusions in psychotic patients and believe that speech illusions may be caused by cognitive processing disorders [22]. They propose that the appearance of speech illusions is due to an imbalance between top-down processing (e.g., expectations and prior knowledge) and bottom-up processing (e.g., sensory input). That is, when internal expectations and external perceptions do not match, top-down processing is activated preferentially [23]. Similarly, Rimvall et al. [24] believe that speech illusions are due to a failure of self-monitoring caused by incorrect matching of internal cognition and external perception, which severely affects bottom-up processing and top-down processing. Hird et al. [25] further verified this opinion through perception experiments. They found that psychotic patients are more likely to perceive noise as spoken words due to the influence of internal cognition, and it is difficult to judge whether neutral statements under noise are positive or negative.

### 1.3 The current study

Through previous research on the neurological and psychological aspects of drug addicts, it has been found that due to damage to certain brain areas and the nervous system, drug addicts may exhibit severe cognitive processing disorders, such as speech illusions and an exaggeration of negative stimuli. However, it is unclear whether drug addicts have difficulties in perceiving communicative functions conveyed through speech and whether their perception of communicative functions under noisy conditions is abnormal. To investigate these questions, this study particularly focused on heroin addicts and conducted a study of communicative function perception in Mandarin Chinese among 25 male heroin addicts and 35 male healthy controls. The study aimed to examine the perception patterns of statement, interrogative, and imperative communicative functions among the two groups in environments with no noise and different noise levels (i.e., signal-to-noise ratio [SNR] = 10 and SNR = 0). Based on previous research, two hypotheses were proposed: (1) heroin addicts would exhibit significantly lower accuracy in perceiving communicative functions than the control group; (2) compared to quiet environments, heroin addicts would exhibit further decreased accuracy in perceiving the three communicative functions under noisy conditions. The results of this study not only fill the research gap in speech perception among drug addicts, but also provide guidance for drug addiction recovery and speech rehabilitation therapy, promote innovative research in Chinese-language-related fields, and assist addicts in returning to society and resuming normal lives.

## 2. Methods

The research was approved by the Committee for the Protection of Human Subjects (CPHS) at the Academy of Forensic Science in Shanghai, China. All participants were informed about the study’s purpose, provided with written consent, and received financial compensation after completion of the experiment. Participants were informed that they could withdraw from the experiment at any time should they choose to discontinue their participation. All participants involved in the current study were recruited to participate in this experiment from September and October 2022.

### 2.1 Speech material

As shown in Table 1, this study included a total of 14 target sentences, each containing 6–10 Chinese characters. Each target sentence is natural sentence used in everyday life and can be expressed in three communicative functions, viz., statement, interrogative, and imperative. To ensure the naturalness of the expressions of communicative functions, different scenarios were designed for the interrogative and imperative. Each scenario contained of two rounds of dialogue, with the target sentence being produced in the second round. All speech materials were revised by three native Mandarin speakers until they were considered natural enough.

**Table 1:** Target sentences involved in the perception experiment.

Speaker		Target sentence	Meaning in English	Communicative functions
Male	1	你们最后出场 /ni3/ /mən0/ /tsweɪ4/ /xoʊ4/ /ʈʂʰu1/ /ʈʂʰɑŋ3/	You are the last to appear.	Statement/ Imperative/ Interrogative
	2	阿丽今天不能出院 /a1/ /li4/ /tɕɪn1/ /tʰjɛn1/ /pu4/ /nɤŋ2/ /ʈʂʰu1/ /ɥœn4/	Ali cannot be discharged from the hospital today.	
	3	洗衣液不能超过八毫升 /ɕi3/ /i1/ /jɛ4/ /pu4/ /nɤŋ2/ /ʈʂʰɑʊ1/ /kuɔ4/ /pa1/ /xɑʊ2/ /ʂɤŋ1/	The laundry detergent should not exceed eight milliliters.	
	4	开会期间不能拿出手机 /kʰaɪ1/ /xweɪ4/ /tɕʰi1/ /tɕjɛn1/ /pu4/ /nɤŋ2/ /na2/ /ʈʂʰu1/ /ʂoʊ3/ /tɕi1/	Mobile phones are not allowed to be taken out during the meeting.	
Female	1	这里不能吸烟 /ʈʂɤ4/ /li3/ /pu4/ /nɤŋ2/ /ɕi1/ /jɛn1/	Smoking is not allowed here.	
	2	全体警察必须到场 /tɕʰɥœn2/ /tʰi3/ /tɕɪŋ3/ /ʈʂʰa2/ /pi4/ /ɕy1/ /tɑʊ4/ /ʈʂʰɑŋ3/	All police officers must be present.	
	3	青少年可以适当玩游戏 /tɕʰɪŋ1/ /ʂɑʊ4/ /njɛn2/ /kʰɤ3/ /i3/ /ʂɚ4/ /tɑŋ4/ /wan2/ /joʊ2/ /ɕi4/	Teenagers can play games in moderation.	
	4	全体员工都要参加培训 /tɕʰɥœn2/ /tʰi3/ /ɥœn2/ /kʊŋ1/ /toʊ1/ /jɑʊ4/ /tsʰan1/ /tɕja1/ /pʰeɪ2/ /ɕyn4/	All employees are required to attend the training.	

This study recruited two native speakers of Mandarin Chinese, one male and one female, with a Putonghua Proficiency Level at the first grade B. The target sentences were recorded in a quiet recording studio using a Zoom H5n professional recorder with a sampling rate of 48.0 kHz in 16 bits. Before recording, the two speakers familiarized themselves with the recording materials and conducted a trial reading. Then, they recorded the 14 target sentences in the statement, interrogative, and imperative. Each target sentence was separated by 1 second, and each communicative function was separated by 2 minutes to allow for the recovery of vocal cords, breathing, and reading states. Finally, a total of 84 target sentences (14 target sentences × 3 communicative functions × 2 speakers) were recorded.

To ensure the accuracy of target sentence expression and the validity of research materials, four native Mandarin speakers were recruited to conduct a perception evaluation experiment on all 84 target sentences. After listening to each target sentence, the participants evaluated the communicative function expressed by the target sentence from four options (i.e., statement, interrogative, imperative, and unable to judge). All target sentences with an average perception accuracy rate of over 75% (3 times the chance level [25%]) were considered valid for the experiment. Additionally, to avoid the attention deficit of drug addicts affecting the experiment results [26, 27], the experiment duration was controlled to about 15 minutes to maintain the addicts’ focus during the experiment. Finally, as shown in Table 1, the four target sentences with the highest average perception accuracy rate in each communicative function were selected from each speaker as the materials for the perception experiment.

The Praat software [28] was used to generate Gaussian white noise, and the sound intensity of the selected 24 target sentences was normalized to 70 dB. Then, the 24 target sentences were mixed with Gaussian white noise to create two levels of noise intensity: SNR = 10 and SNR = 0. Under the SNR = 10 condition, Gaussian white noise was mixed with target sentences 1 and 2, while under the SNR = 0 condition, Gaussian white noise was mixed with target sentences 3 and 4. Finally, a total of 48 speech stimuli (24 No noise + 12 SNR10 + 12 SNR0) were obtained as the speech materials for the perception experiment.

### 2.2 Participants of perception experiment

We conducted a preliminary power analysis using the *pwr* package in R software [29, 30] to determine the required number of participants. The results indicated that a sample size of more than 20.03 per group would be needed to achieve a statistical power of 0.80 (at a significance level of 0.05) in order to detect a large difference among the groups. The effect size, defined as Cohen’s d, was set at 0.8 to anticipate a large between-groups difference [31].

This study recruited a total of 60 male participants for the perception experiment, including 25 male heroin addicts and 35 healthy controls. The heroin addicts were all recruited from a compulsory drug rehabilitation center in China, with a duration of less than 2 months in the rehabilitation center. All participants voluntarily participated in the experiment and signed an informed consent form.

The mean age of the control group was 35.74 years (sd = 11.35), and the mean age of the heroin addicts was 37.14 years (sd = 9.72). The average duration of drug use among the heroin addicts was 7.25 years (sd = 6.14) since their first exposure to the drug. It should be noted that all heroin addicts included in the study have not undergone any periods of drug abstinence during their drug use. All participants were right-handed and had no history of speech or hearing impairments.

### 2.3 Procedure

**Fig 1:**
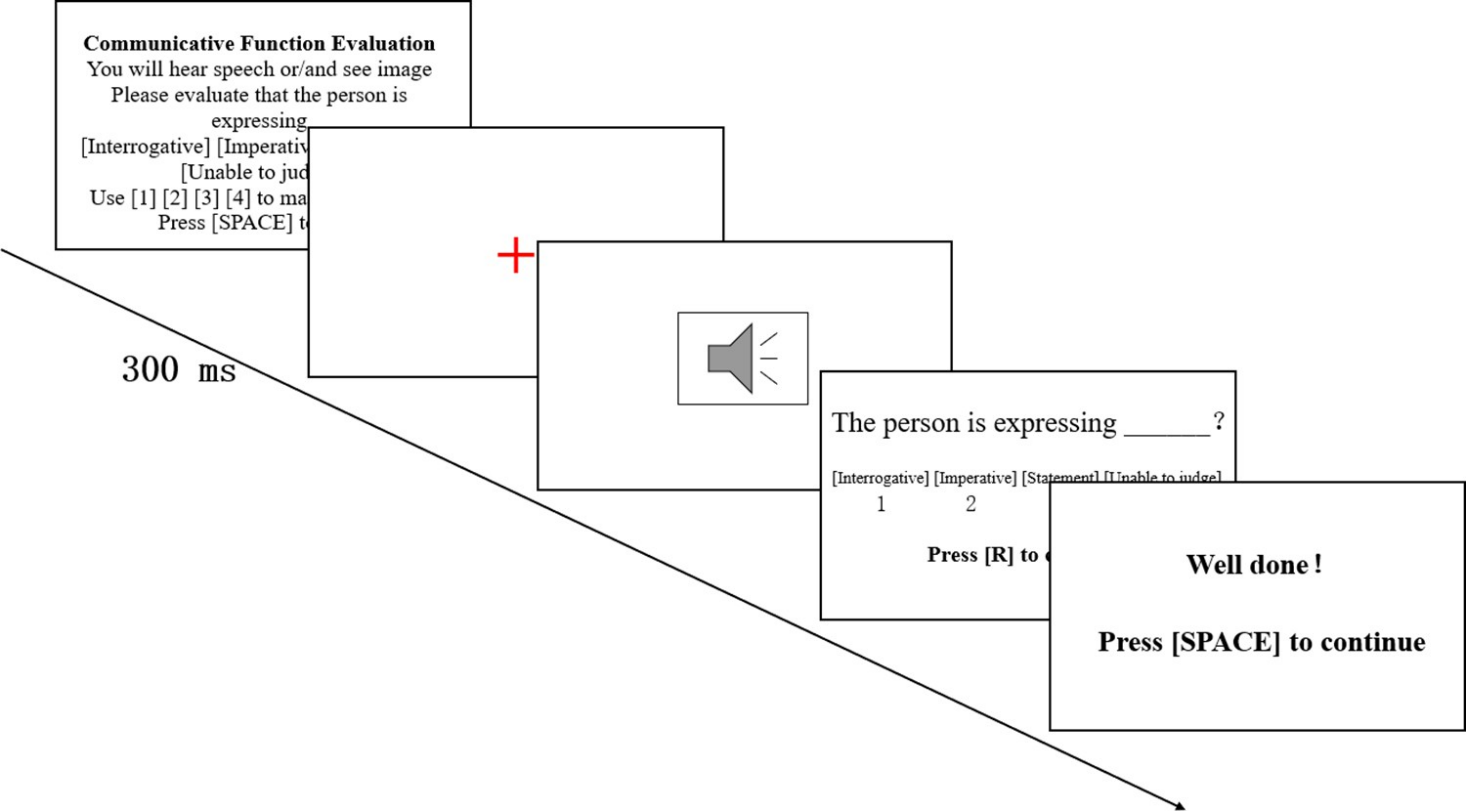
The procedure of perception experiment.

The perception experiment was conducted in a quiet room, and all participants wore professional headphones (Sennheiser HD650 and Audio-Technica ATH-M70x) for the experiment. The PsychoPy software (v2022.2.0) [32] was used to present all 48 speech stimuli in a fixed random order. The experimental procedure is shown in Fig 1. Before the experiment, participants adjusted the distance between the laptop screen and themselves according to their own visual acuity and carefully read the experiment instructions.

During the experiment, a red fixation point "+" appeared in the center of the screen for 300ms, followed by the presentation of the speech stimulus. Participants were required to evaluate the communicative function expressed by the stimulus or listen to the stimulus again before making a selection. Four options were provided for the perception experiment: statement, interrogative, imperative, and unable to judge. Prior to the formal experiment, participants completed a practice session to familiarize themselves with the experimental procedure. During the formal experiment, participants could take breaks at any times.

### 2.4 Statistical analysis

In order to investigate whether there were differences in the perception distribution and response time of the three communicative functions between the addicts and the control group under different noise levels, a linear mixed-effect model was built based on each speech stimulus using the *lme4* package [33] and the *lmerTest* package [34] in R software [30].

Further, to investigate the effects of drug type and noise level on the perception of communicative functions, the correct or incorrect perception judgment for each speech stimulus was firstly recoded as 0 (incorrect) and 1 (correct), and a generalized logistic regression model was built using the *afex* package [35].

In each model, the communicative function (statement, interrogative, and imperative), drug type (control group and heroin addicts), and noise level (No noise, SNR = 10, and SNR = 0) were fixed effects, while the participant and age were random effects. Finally, the *lsmeans* package [36] was used to conduct Tukey *post hoc* tests on the significant effects. To evaluate the effect sizes of the statistical analyses, this study reported the odds ratio (OR) as the effect size.

## 3. Results

**Fig 2:**
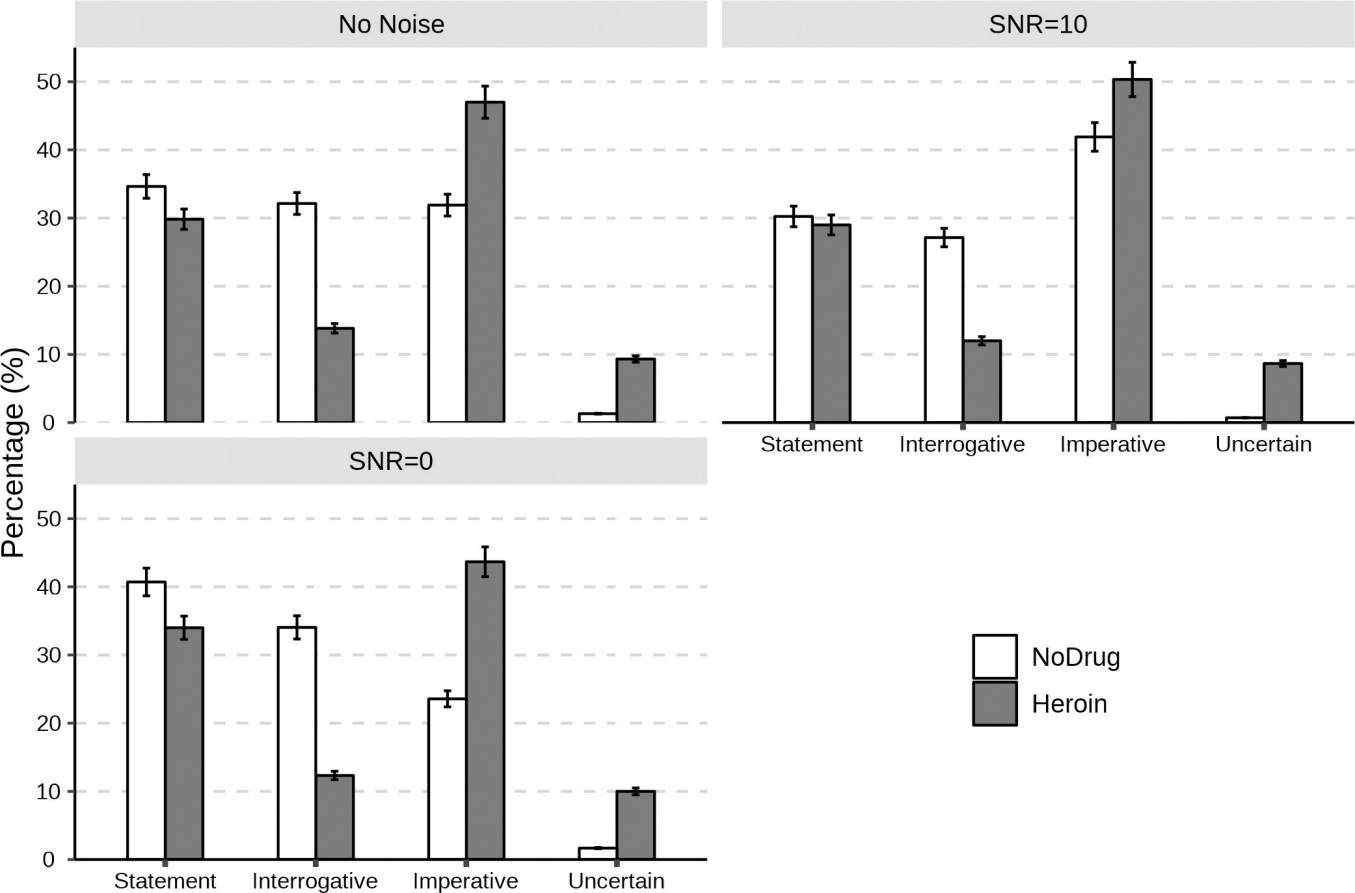
The distribution (error bars indicate 95%CI) of the perception of the speech conveying three communicative functions by heroin addicts and the control group under different noise levels (i.e., No Noise, SNR = 10, and SNR = 0).

The perception distribution of the speech that conveys the three communicative functions by heroin addicts and the control group under different noise levels is shown in Fig 2, and the percentage values are shown in Table 2. The experiment found that regardless of the noise level, heroin addicts judged around 50% of the speech stimuli as imperative, while their selection of interrogative was only about 12%. Compared with heroin addicts, the perception distribution of the speech stimuli by the control group was more even. In addition, the proportion of choices that they were unable to judge made by heroin addicts was also about 10% higher than that of the control group.

**Table 2:** The perception distribution (%) of the speech conveying the three communicative functions by heroin addicts and the control group under different noise levels (i.e., No noise, SNR = 10, and SNR = 0).

Drug type	Noise level	Distribution (%)			
		Statement	Interrogative	Imperative	Unable to judge
Control group	No noise	34.64	32.14	31.9	1.31
	SNR = 10	30.24	27.14	41.9	0.71
	SNR = 0	40.71	34.05	23.57	1.67
Heroin	No noise	29.83	13.83	47	9.33
	SNR = 10	29	12	50.33	8.67
	SNR = 0	34	12.33	43.67	10

**Fig 3:**
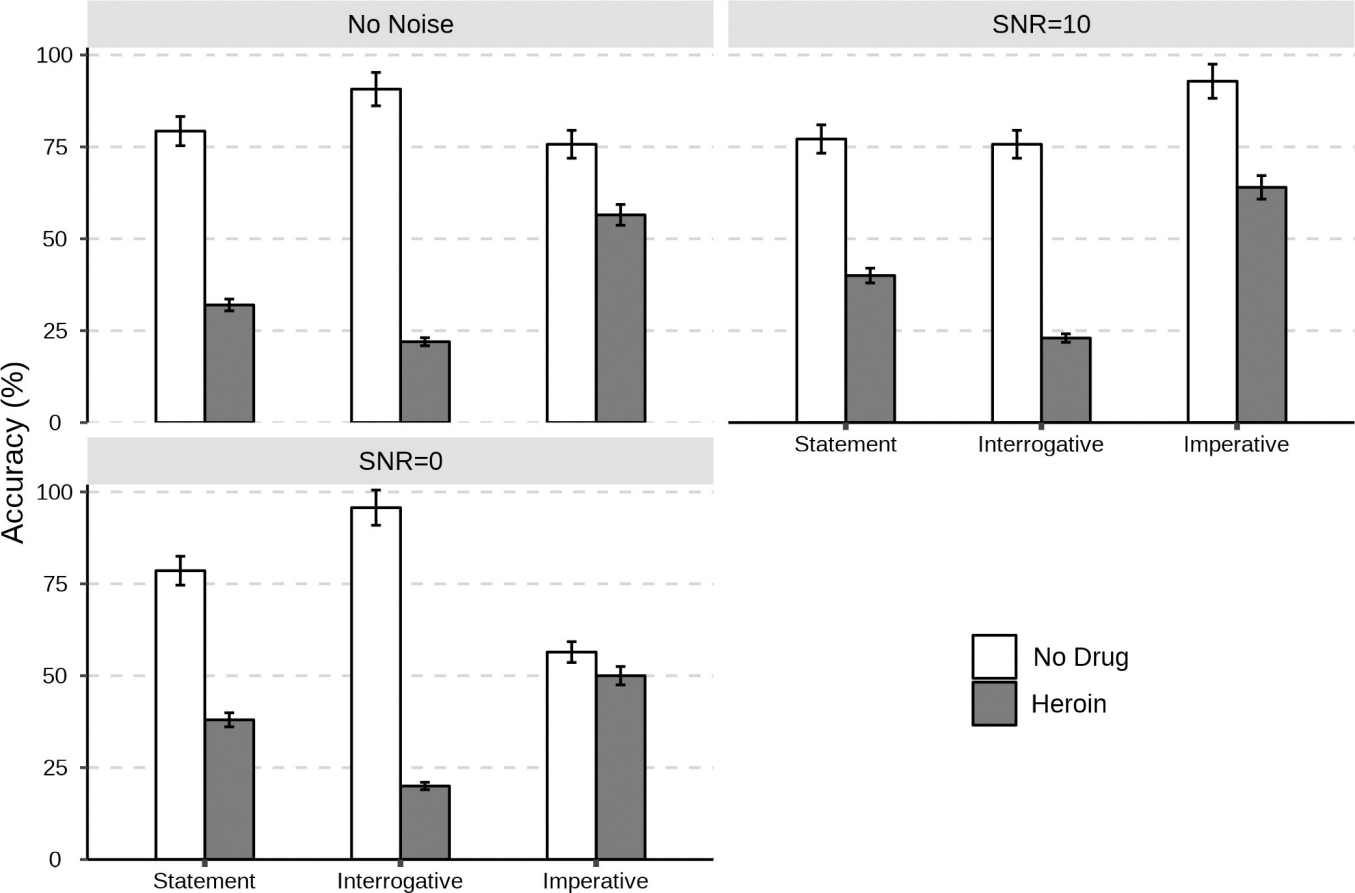
The perception accuracy (error bars indicate 95%CI) of the three communicative functions by heroin addicts and the control group under different noise levels (i.e., No Noise, SNR = 10, and SNR = 0).

The perception accuracy of the three communicative functions by heroin addicts and the control group under the three noise levels is shown in Fig 3. The average perception accuracy of the control group for the three communicative functions was 80.66%, while the average perception accuracy of heroin addicts was 38%. As shown in Table 3, under different noise levels, the perception accuracy of the control group for the three communicative functions was approximately between 75% and 95%, significantly higher than that of heroin addicts. As the noise level increased, the perception accuracy of the control group for the communicative functions changed slightly. The perception accuracy of heroin addicts for statement and interrogative did not change much, while the perception accuracy for imperative decreased somewhat under high noise levels (SNR = 0). It is worth noting that the perception accuracy of heroin addicts for imperative was between 50% and 65%, higher than that of statement and interrogative. However, for interrogative, the perception accuracy of heroin addicts was only about 22%, lower than the chance level of this experiment (1/4 = 25%).

**Table 3:** The perception accuracy (%) and response time (second) of the three communicative functions by heroin addicts and the control group under different noise levels (i.e., No noise, SNR = 10, and SNR = 0).

		Control group			Heroin		
		No noise	SNR = 10	SNR = 0	No noise	SNR = 10	SNR = 0
**Perception**	**Statement**	79.29	77.14	78.57	32	40	38
**accuracy**	**Interrogative**	90.71	75.71	95.71	22	23	20
**(%)**	**Imperative**	75.71	92.86	56.43	56.5	64	50
**Response**	**Statement**	2.01	1.58	2.48	3.1	2.85	4.42
**Time**	**Interrogative**	1.21	1.91	1.19	2.58	4.55	2.07
**(s)**	**Imperative**	2.49	1.54	1.93	4.22	3.66	2.92

For response time, the control group showed an average response time of 1.82 seconds, while the heroin group showed an average response time of 3.37 seconds. As shown in Table 3, the heroin group exhibited significantly longer response times than the control group across all communicative functions, regardless of the noise conditions.

As shown in Table 4, the results of the linear mixed-effect model revealed significant main effects of "communicative function" and "drug type", significant two-way interaction effects of "drug type × communicative function" and "communicative function × noise level", and a significant three-way interaction effect of "drug type × noise level × communicative function" were found for perception distribution. For response time, significant main effects of "communicative function" and "drug type", significant two-way interaction effects of "communicative function × noise level", and a significant three-way interaction effect of "drug type × noise level × communicative function" were found.

**Table 4:** The results of linear mixed effect model analysis on the perception distribution and response time of the speech conveying the three communicative functions between heroin addicts and control group with communicative function, drug type and noise level as fixed factors.

	Factors	df	*F*	*p*
Perception distribution	Communicative function	2	455.72	**< 0.001**
	Drug type	1	76.12	**< 0.001**
	Noise level	2	2.41	0.09
	Drug type × Communicative function	2	254.38	**< 0.001**
	Drug type ×Noise level	2	0.07	0.94
	Communicative function × Noise level	4	6.71	**< 0.001**
	Drug type × Noise level ×Communicative function	4	3.02	**0.02**
Response time	Communicative function	2	10.58	**< 0.001**
	Drug type	1	32.98	**< 0.001**
	Noise level	2	0.45	0.09
	Drug type × Communicative function	2	0.02	0.98
	Drug type ×Noise level	2	2.40	0.09
	Communicative function × Noise level	4	19.74	**< 0.001**
	Drug type × Noise level ×Communicative function	4	3.41	**0.008**

When the higher-order interaction effects are significant, post-hoc tests for main effects and lower-order interaction effects may not be conducted. The Tukey *post hoc* test for the three-way interaction effect of "drug type × noise level × communicative function" on perception distribution revealed significant differences in the perception of the speech conveying the three communicative functions between heroin addicts and the control group under the three noise levels (no noise: statement *β* = -0.36, *SE* = 0.07, *z* = -5.44, *p* < 0.001; interrogative *β* = 1.17, *SE* = 0.07, *z* = 17.51, *p* < 0.001; imperative *β* = 0.19, *SE* = 0.07, *z* = 2.81, *p* = 0.005. SNR10: statement *β* = -0.20, *SE* = 0.09, z = -2.21, *p* = 0.03; interrogative *β* = 0.90, *SE* = 0.09, *z* = 10.15, *p* < 0.001; imperative *β* = 0.29, *SE* = 0.09, *z* = 3.28, *p* = 0.001. SNR = 0: statement *β* = -0.33, *SE* = 0.09, *z* = -3.75, *p* < 0.001; interrogative *β* = 1.20, *SE* = 0.09, *z* = 13.52, *p* < 0.001; imperative *β* = 0.19, *SE* = 0.09, *z* = 2.08, *p* = 0.04). Besides, the results of post hoc test on response time revealed that heroin addicts exhibited longer response time than the control group for all three communicative functions under the three noise levels (no noise: statement *β* = 1.14, *SE* = 0.35, *z* = 3.23, *p* = 0.002; interrogative *β* = 1.41, *SE* = 0.35, *z* = 3.99, *p* < 0.001; imperative *β* = 1.53, *SE* = 0.35, *z* = 4.33, *p* < 0.001. SNR10: statement *β* = 1.19, *SE* = 0.44, z = 2.72, *p* = 0.007; interrogative *β* = 2.25, *SE* = 0.43, *z* = 5.21, *p* < 0.001; imperative *β* = 2.12, *SE* = 0.43, *z* = 4.91, *p <* 0.001. SNR = 0: statement *β* = 2.09, *SE* = 0.44, *z* = 4.79, *p* < 0.001; interrogative *β* = 0.88, *SE* = 0.43, *z* = 2.04, *p* = 0.04; imperative *β* = 0.91, *SE* = 0.44, *z* = 2.07, *p* = 0.04).

As shown in Table 5, the results of the generalized linear regression model revealed significant main effects of "communicative function" and "drug type", significant two-way interaction effects of "drug type × communicative function" and "communicative function × noise level", and a significant three-way interaction effect of "drug type × noise level × communicative function" on the perception accuracy of the three communicative functions between heroin addicts and the control group.

**Table 5:** The results of generalized linear regression model analysis on the perception accuracy of the three communicative functions between heroin addicts and control group with communicative function, drug type and noise level as fixed factors.

Factors	df	χ^2^	*p*
Communicative function	2	16.81	**< 0.001**
Drug type	1	93.17	**< 0.001**
Noise level	2	2.43	0.30
Drug type × Communicative function	2	95.28	**< 0.001**
Drug type ×Noise level	2	0.78	0.68
Communicative function × Noise level	4	56.06	**< 0.001**
Drug type × Noise level ×Communicative function	4	34.85	**< 0.001**

A Tukey *post hoc* test was conducted on the three-way interaction effect of "drug type × noise level × communicative function". It was found that under the conditions of no noise and SNR = 10, heroin addicts showed significantly lower accuracies than the control group in the perception of all three communicative functions (no noise: statement *β* = -2.29, *SE* = 0.28, *z* = -8.23, *p* < 0.001, OR = 8.09; Interrogative: *β* = -3.87, *SE* = 0.33, *z* = -11.88, *p* < 0.001, OR = 34.20; Imperative: *β* = -0.94, *SE* = 0.27, *z* = -3.54, *p* < 0.001, OR = 2.40. SNR 10: statement *β* = -1.76, *SE* = 0.34, *z* = -5.13, *p* < 0.001, OR = 5.02; Interrogative: *β* = -2.56, *SE* = 0.37, *z* = -7.01, *p* < 0.001, OR = 10.31; Imperative: *β* = -2.15, *SE* = 0.43, *z* = -4.98, *p* < 0.001, OR = 7.25). For SNR = 0, heroin addicts showed significantly lower accuracies than the control group in perception of statement and interrogative (statement: *β* = -1.94, *SE* = 0.35, *z* = -5.59, *p* < 0.001, OR = 5.93; Interrogative: *β* = -4.86, *SE* = 0.53, *z* = -9.26, *p* < 0.001, OR = 86.20), but no significant difference was found in perception of imperative (*β* = -0.23, *SE* = 0.32, *z* = -0.72, *p* = 0.47, OR = 1.29).

## 4. Discussion

This study aims to investigate the perception of communicative functions (i.e., statement, interrogative, and imperative) in Mandarin Chinese under different noise levels (i.e., No Noise, SNR = 10, and SNR = 0) between male heroin addicts and the healthy controls. The results show that the average perception accuracy for the three communicative functions of the control group is significantly higher than that of the heroin addicts (80.66% vs. 38%). The perception of communicative functions in heroin addicts under different noise levels differs significantly from the control group, and their selection of imperative is about 50%, while their selection of interrogative is only about 12%. Under low noise levels (i.e., no noise and SNR = 10), there is a significant difference between heroin addicts and the control group in the recognition of the three communicative functions. Under high noise levels (i.e., SNR = 0), there is a significant difference between heroin addicts and the control group in the recognition of statement and interrogative, but there is no significant difference in the perception of imperative. Furthermore, heroin addicts exhibited longer response time than the control group for all three communicative functions regardless of the noise levels.

The current study finds that heroin addicts have more responses for imperative and fewer responses for interrogative in the perception experiment. This finding partially confirms previous research on the neural cognitive processing of drug addicts, namely that drug addicts are more likely to experience negative emotions such as stress and anxiety [16, 17, 37–39]. The consistent results of previous research and the current study in terms of neural cognition and speech perception indicate the need for speech rehabilitation treatment during drug detoxification treatment for heroin addicts. In speech rehabilitation or social communication with addicts, it is necessary to avoid negative "misunderstandings" of communicative functions, which can also help reduce the possibility of negative behaviors such as relapse and drug abuse by addicts. However, whether the findings of this paper on heroin addicts can be generalized to other drug addicts requires more extensive research in the future to verify.

Secondly, the finding of the current study that the perception accuracy of communicative functions in heroin addicts is lower than that of the control group confirms the hypothesis (1) proposed in this paper. This result indicates that heroin affects the perception of communicative functions in daily speech communication, which may lead to "misunderstandings" in speech communication. The findings of the present study can have several clinical implications for heroin addicts. Firstly, it can inform the development of targeted interventions and therapies to improve their communication skills (e.g., recognizing interrogative expression) and enhance their overall social functioning. By identifying specific areas of difficulty, healthcare professionals can tailor interventions to address these challenges and help individuals with heroin addiction better understand and interpret communicative functions in Mandarin speech. Besides, examining the perception of Mandarin speech in this population can shed light on the impact of drug addiction on cognitive function, language processing, and communication abilities. This information can help healthcare providers in assessing and monitoring the cognitive and linguistic status of individuals with heroin addiction, facilitating early detection of potential deficits and appropriate intervention strategies.

However, with the increase of noise level, there is no trend of gradual decrease in the perception accuracy of statement and interrogative in heroin addicts and the control group, except for the perception of imperative, which is reduced to about 50% in both groups under high noise (SNR = 0). This finding contradicts hypothesis (2) proposed in this paper, that the perception accuracy of communicative functions in both groups does not decrease with the increase of noise level except for imperative. The reason for this phenomenon may be that the speech perception of heroin addicts under noise conditions is not greatly affected, and their lower perception accuracy is not caused by noise but by abnormal neural cognitive mechanisms related to communicative function perception. This phenomenon also deserves further confirmation and discussion in more in-depth neural cognitive research in the future.

It is worth noting that regardless of whether there is noise or not, heroin addicts’ perception accuracy of imperative is higher than that of the other two communicative functions. This may be because heroin addicts are more inclined to perceive the auditory stimulus as an imperative, which to some extent increases the perception accuracy of imperative.

In addition, the finding that the perception accuracy of interrogative in heroin addicts is only about 22%, which is lower than the chance level (25%) of the perception experiment (see Table 3). This result indicates that heroin addicts are unable to recognize the interrogative function conveyed in speech. In fact, Mandarin Chinese mainly relies on pitch changes to express the interrogative function in the absence of modal particles (such as "/ma/") where the pitch curve shows a continuous rise and reaches the highest point at the end of the sentence. Previous research has found that some drugs can cause reversible changes in the perception of pitch, such as carbamazepine and levetiracetam used to treat epilepsy, which can cause reversible pitch perception disorders in drug users [40–42]. In addition, Groot [43] also reported similar cases in which a patient with depression had a significant decrease ability in pitch perception after taking lithium, desipramine, and oxazepam. Groot believed that pitch perception involves the auditory cortex and adjacent areas in the temporal lobe [44, 45], and the effects of these drugs on the corresponding areas may be the reason for the disorder in pitch perception. Therefore, the current paper speculates that heroin may have an impact on the auditory cortex and temporal lobe related to pitch perception, which makes addicts insensitive to pitch changes and ultimately impacts the recognition of interrogative. This hypothesis needs further in-depth neural cognitive research in the future to validate.

It is also interesting to note that the heroin addicts exhibited longer response time than the control group for all three communicative functions. Previous research has shown that certain drugs, such as heroin, cocaine, and amphetamine, can enhance memory and cognitive functions [46–48]. However, this study found that heroin did not improve processing speed and accuracy for communicative functions as expected. In fact, Wood et al. [49] have found that the impact of drugs on cognitive processing follows an inverted U-shaped curve, with moderate usage enhancing processing while excessive usage leading to cognitive impairments. Similarly, some scholars argue that long-term and excessive drug use can cause severe damage to cognitive functions [50, 51]. The heroin addicts recruited for this study had an average addiction duration of over 7 years, which to some extent demonstrates the negative impact of long-term drug addiction on cognition.

Research has shown that craving can lead to decreased processing speed and accuracy, or cognitive inhibition, among individuals with drug addiction [52–54]. Indeed, it is widely acknowledged that drug craving can impact cognitive functioning. Scholars have found that craving in drug addicts can result in increased stress, anxiety, and negative emotions [55–57]. Additionally, some researchers have proposed that drug craving can cause attentional bias, making individuals more susceptible to drug-related stimuli. For example, Field et al. [58] found that cannabis addicts with high levels of craving exhibited a significant attentional bias towards cannabis-related words in a visual probe task, while those with low levels of craving did not. Similarly, Lubman et al. [59] found that opiate addicts displayed attentional bias towards drug-related images. Using a modified visual probe task, Franken [60] demonstrated that variation in cocaine craving was associated with attentional bias towards cocaine-related words, with higher levels of craving corresponding to stronger attentional biases towards drug-related stimuli. In the context of speech perception for communicative functions, drug craving among heroin addicts may result in attentional bias, which presents an intriguing phenomenon worthy of further investigation. Some researchers argue that reducing craving can contribute to the restoration of cognitive functioning in individuals with addiction [61]. Therefore, understanding the extent of drug craving in drug addicts and finding effective ways to mitigate craving are of significant importance for the recovery of communicative function perception in individuals with drug addiction.

There are several limitations of this study that need to be noted. Firstly, this study recruited 25 male heroin addicts, and future research could expand the sample size and examine other drug types and genders. Secondly, this study examined the effects of heroin addiction on communicative functions in Mandarin Chinese through a speech perception experiment and preliminarily found significant differences in communicative function perception between heroin addicts and the control group. However, the underlying neural and cognitive mechanisms that cause these differences are still unclear and require further investigation through neural cognitive research. Lastly, whether the changes in perception of communicative function observed in drug addicts will affect their speech production needs to be explored in future research.

## 5. Conclusion

This study investigated the perception of communicative functions in Mandarin Chinese among male heroin addicts and found that the perception accuracy of communicative function among heroin addicts was lower than that of the control group, except for imperative perception under high noise conditions. Additionally, significant differences in communicative function perception were observed between heroin addicts and the control group under different levels of noise. Furthermore, heroin addicts exhibited better recognition rate of imperative but poorer recognition rate of interrogative. These findings not only fill the research gap in communication function perception among drug addicts in Mandarin Chinese but also enhance our understanding of the effects of drugs on speech perception. Moreover, this study provides a theoretical foundation for speech rehabilitation for drug addicts and has significant practical value in helping them return to society and resume normal life.

## Supporting information

S1 File (CSV)
